# Chronic Contractile Dysfunction without Hypertrophy Does Not Provoke a Compensatory Transcriptional Response in Mouse Hearts

**DOI:** 10.1371/journal.pone.0158317

**Published:** 2016-06-30

**Authors:** Scot J. Matkovich, David R. Grubb, Julie R. McMullen, Elizabeth A. Woodcock

**Affiliations:** 1 Center for Pharmacogenomics, Department of Internal Medicine, Washington University School of Medicine, St. Louis, MO, United States of America; 2 Baker IDI Heart and Diabetes Institute, Melbourne, Australia; Texas A& M University Health Science Center, UNITED STATES

## Abstract

Diseased myocardium from humans and experimental animal models shows heightened expression and activity of a specific subtype of phospholipase C (PLC), the splice variant PLCβ1b. Previous studies from our group showed that increasing PLCβ1b expression in adult mouse hearts by viral transduction was sufficient to cause sustained contractile dysfunction of rapid onset, which was maintained indefinitely in the absence of other pathological changes in the myocardium. We hypothesized that impaired contractility alone would be sufficient to induce a compensatory transcriptional response. Unbiased, comprehensive mRNA-sequencing was performed on 6 biological replicates of rAAV6-treated blank, PLCβ1b and PLCβ1a (closely related but inactive splice variant) hearts 8 weeks after injection, when reduced contractility was manifest in PLCβ1b hearts without evidence of induced hypertrophy. Expression of PLCβ1b resulted in expression changes in only 9 genes at FDR<0.1 when compared with control and these genes appeared unrelated to contractility. Importantly, PLCβ1a caused similar mild expression changes to PLCβ1b, despite a complete lack of effect of this isoform on cardiac contractility. We conclude that contractile depression caused by PLCβ1b activation is largely independent of changes in the transcriptome, and thus that lowered contractility is not sufficient in itself to provoke measurable transcriptomic alterations. In addition, our data stress the importance of a stringent control group to filter out transcriptional changes unrelated to cardiac function.

## Introduction

Heart failure, a condition wherein the pumping ability of the heart is severely compromised, develops as the end result of many different cardiac diseases. The prevalence of heart failure worldwide is increasing and it contributes substantially to the rising cost of health care. Despite this, current treatment options are largely limited to amelioration of symptoms. The failing heart exhibits a number of characteristics that contribute to pump failure. Some of these involve non-myocytes, especially fibroblasts that are instrumental in remodelling the heart [1] but some changes occur within the myocytes themselves [2]. Myocytes from failing hearts exhibit structural changes as well as alterations in intracellular signaling pathways and calcium handling [3–5]. Importantly, the calcium content of the sarcoplasmic reticulum (SR) is lowered, partly due to heightened calcium leak into the cytosol mediated by ryanodine receptors and partly due to reduced SR calcium uptake via the sarcoendoplasmic reticulum calcium ATPase (SERCA) [2, 6]. Failing myocytes characteristically show lowered expression of the SR calcium pump SERCA2a encoded by *Atp2a2* [7] along with reduced phosphorylation of phospholamban (PLN), which further depresses SERCA2a activity [8]. PLN is phosphorylated by protein kinase A downstream of β-adrenergic receptor activation and also by calcium-calmodulin regulated kinase II (CaMKIIδ) at a neighbouring site [9]. These PLN phosphorylations disinhibit SERCA and enhance lusitropy; therefore the lowered β-receptor expression and activity that characterize the failing myocardium likely contribute to SR calcium depletion [10].

**Fig 1:**
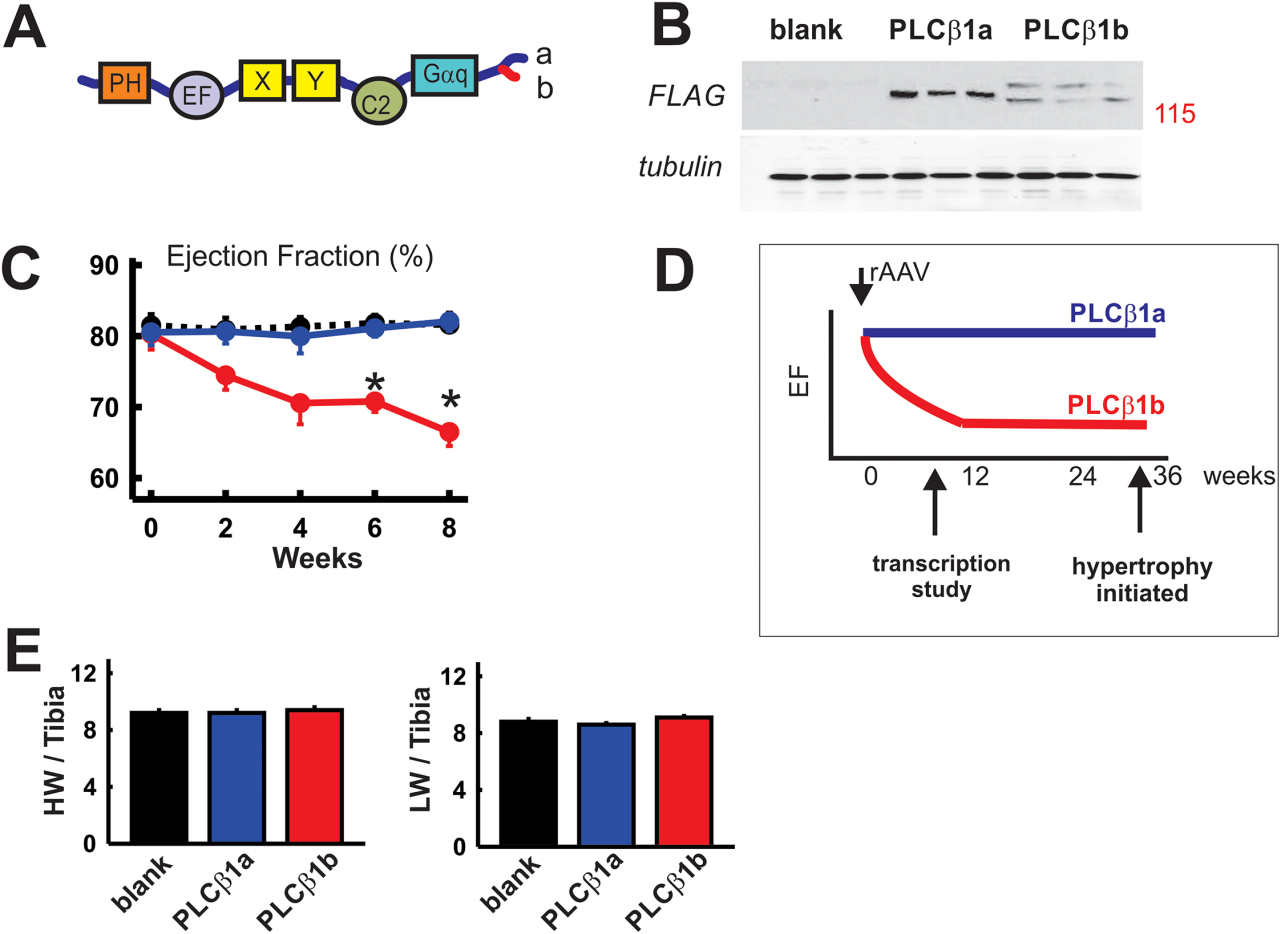
Expression of PLCβ1b in mouse heart for 8 weeks causes contractile dysfunction. **A**. Comparative structures of PLCβ1b and PLCβ1a. PH is PH domain, EF is EF hand domain, X and Y form the catalytic site, C2 is C2 domain, Gαq is the Gαq binding region. **B.** Western blots using anti-FLAG (Anti-DYKDDDDK rabbit polyclonal antibody Abnova, 1/5000) and anti-β-tubulin antibody (1/1000 (abcam, ab6046)) showing FLAG-PLCβ1b and -PLCβ1a expressions in left ventricle 8 weeks after rAAV6-mediated delivery, 3 samples per group. **C.** Time course of contractility changes measured as ejection fraction (%) in hearts following treatment with rAAV6 expressing PLCβ1b, PLCβ1a or blank control. Values are ejection fraction (%), mean ± SEM, n = 6. * p<0.01 relative to PLCβ1a or blank; data were analysed using a 2 way ANOVA for repeat measurements, as described previously (14). *Red*, PLCβ1b; *blue*, PLCβ1a; *black*, blank control. **D**. Diagram showing the time frame of changes in function in relation to the 8 week time point used in the current study [14]. **E**. Organ responses to PLCβ1b or PLCβ1a expression. *Left panel*: heart wt/tibia (mg/mm). *Right panel*; lung wt/tibia mg/mm. Values are mean ± SEM, n = 6.

In addition to these considerations, recent studies in our laboratory have identified another factor that contributes to contractile depression in the failing hearts. Failed myocardium from humans and experimental animals showed heightened expression and activity of phospholipase Cβ1b (PLCβ1b) [11], one of the splice variants of PLCβ1 [12] that initiates signaling responses downstream of Gq-coupled receptor activation [13]. Our studies showed that increasing PLCβ1b expression in adult mouse hearts by viral transduction was sufficient to cause rapidly developing, sustained contractile dysfunction [14], which lasted for several months in the absence of other pathological changes in the myocardium [14] (time course depicted diagrammatically in Fig 1). Further evidence pointing to the significance of the contribution of heightened PLCβ1b to pathology was provided by a more recent study [15], which showed that inhibiting PLCβ1b selectively prevented or reversed contractile dysfunction following pressure overload induced by trans-aortic constriction. Along with the improved contractility, the PLCβ1b-specific inhibitory mini-gene reduced hypertrophy and prevented premature death [15].

Chronically heightened PLCβ1b expression results in compensatory hypertrophy and some fibrosis only after a prolonged period (36 weeks) [14]. In agreement with this, transcriptional activation of several ‘classical’ marker genes associated with hypertrophy and fibrosis was detected at 36 weeks, but not at earlier time points. In contrast to the signaling mechanisms responsible for the development of hypertrophy, it is not known whether transcriptional events can be initiated specifically by low contractility, either as causes or consequences of contractile dysfunction. PLCβ1b-induced contractile dysfunction, prior to the onset of hypertrophy, provides the possibility of examining whether there are transcriptional responses associated with chronically depressed contractility in the absence of other changes related to the progression to heart failure. Importantly, transduction of the closely related splice variant PLCβ1a had no effect on contractility [14], or on any other discernible response when expressed in mouse hearts at similar levels to PLCβ1b [16] and thus PLCβ1a provides a stringent control for all studies involving PLCβ1b.

The literature contains many examples of transcriptome studies undertaken using hypertrophied or failed myocardium from humans and animal models [17–19]. Such studies have identified genes associated with cellular growth, fibrosis and remodelling as well as genes associated with calcium handling. Many of these identified genes could contribute to contractile depression, singly or in combination, but such studies raise the issue of which transcriptional changes are primarily causative and which are secondary to remodelling or to the loss of contractile function. Contractile dysfunction caused by PLCβ1b does not induce other changes associated with the progression to failure until a much later time point, and thus provides a unique opportunity to examine transcriptional changes associated directly with low contractile function. Such transcriptional changes could be either causative of dysfunction or secondary to the chronically low contractility. With this in mind, we undertook a comprehensive mRNA-sequencing study of mice with chronically low contractile function due to PLCβ1b overexpression, hypothesizing that reduced cardiac output alone would be sufficient to provoke at least a partly compensatory transcriptional response. We used a time point early in the response to avoid compensatory hypertrophy observed after several months of treatment [14] (Fig 1).

## Methods

All experiments were conducted in accordance with the Australian Code of Practice for ‘The Care and Use of Animals for Scientific Purposes’ of the National Health and Medical Research Council of Australia and all studies were approved by the Alfred Medical Research and Education Precinct Animal Ethics Committee (#1362/2013). Mice used were C57BL/6 obtained from the Animal Resources Centre, Perth.

### Expression of PLCΒ1b or PLCΒ1a in mouse hearts

rAAV6-PLCβ1b, rAAV6-PLCβ1a or rAAV6-blank (control virus with a promoter but no protein product [20]) were prepared as described previously [14]. Male mice, 8 weeks of age, were injected with rAAV6-FLAG-PLCβ1b, rAAV6-FLAG-PLCβ1a or rAAV6-blank intravenously (IV) via the tail vein at a vector dose of 3 x 10^10^ vg/g. This dose has been shown to produce heightened expression of FLAG-PLCβ1b or FLAG-PLCβ1a in all chambers of the heart in all animals [14]. Nine (9) weeks after viral delivery (8 weeks after PLCβ1b/PLCβ1a expression) the animals underwent echocardiographic studies to evaluate contractile function, followed by humane culling, removal of hearts and dissection of the left ventricle (LV) for RNA extraction and western blotting. Extracted RNA subsequently was used for mRNA sequencing (mRNA-Seq) analysis. Western blotting using anti-FLAG and anti-tubulin antibodies to confirm expression in all hearts used for sequencing was performed exactly as described previously (14).

### Measurement of contractile function

In the current study, contractile function was assessed as ejection fraction determined by echocardiography. Anesthesia was maintained with 1.7% isoflurane. Echocardiography was performed using a Philips iE33 ultrasound machine and a 15 MHz linear-transducer. After a short-axis 2-D image of the LV at the level of the papillary muscles was obtained, 2-D guided M-mode images were acquired digitally at a sweep speed of 133 mm/s. Ejection fraction [EF = (LVIDd^3^ –LVIDs^3^)/LVIDd^3^, where LVIDd/s is the left ventricular internal diameter at systole ‘s’ or diastole ‘d’].

### RNA-Seq methods for AAV6-PLCβ1a/b cardiac analysis

Total RNA from mouse heart left ventricle was prepared by Trizol extraction from flash-frozen tissue. Six (6) biological replicates were used for each of rAAV6-blank, rAAV6-PLCβ1a and rAAV6-PLCβ1b conditions. Ribosomal RNA depletion along with RNA fragmentation and conversion to paired-end, strand-specific Illumina sequencing libraries were performed with Illumina TruSeq preparation kits. Sequencing reads of 76 nt in length were obtained from multiplexed libraries on an Illumina NextSeq instrument at the Ramaciotti Centre for Genomics, Sydney, Australia with (3.02 ± 0.11) * 10^7^ paired reads per heart (mean ± SEM). Reads were aligned to the mouse transcriptome represented in the Illumina iGenomes mm10 UCSC release using Tophat [21], achieving a read depth of (1.84 ± 0.07) * 10^7^ paired reads per heart (mean ± SEM); this represents a mean alignment to the transcriptome of 61%. All mRNA-sequencing reads and transcriptome-aligned read counts have been deposited in the NCBI GEO with accession number GSE73909.

### Statistical and informatic procedures

For ejection fraction measurements in Fig 1C, data were analyzed using a 2 way ANOVA for repeat measurements, as described previously [14]. For the newly performed RNAseq measurements in this study, the union mode of HTSeq [22] was used in a strand-specific manner to quantitate reads pertaining to each mRNA. These data were used as input to the DESeq package which adjusted for library depth, performed differential expression calculations, and derived false discovery rates (FDR). Similar findings were made when the HTSeq-quantitated reads were used as input to edgeR for calculating differential expression [23]. For previous RNAseq studies [24–27] employing non-strand-specific mRNA reads, the union mode of HTSeq [22] was used to re-analyze raw RNAseq reads, newly aligned to the mm10 UCSC genome as above, without taking strand identity into account. DESeq was again employed for differential expression calculations.

### Comparison of PLCβ1a / PLCβ1b RNA-Seq data with existing RNA-Seq data sets

Data sets for rAAV6-mediated adult cardiac expression of PLCβ1a and PLCβ1b were compared with existing RNA-Seq datasets of αMHC/*Myh6*-driven expression of Gαq [24–26] or of full-length PKCα cDNA in adult mouse hearts [27] commencing in the neonatal period. Although PKCα expression was under the control of the doxycycline-suppressible tet-off Myh6 promoter, doxycycline was never administered to dams or pups and thus transgene activity commenced at birth, as for Gαq. Only mRNAs with a cardiac abundance of FPKM ≥3 were included, in accordance with the design of the original studies. In total, 781 individual mRNAs exhibited significant difference between PLCβ1a/b and blank virus, between Gαq and wt controls (derived specifically from [24]), or between PKCα and tet-off (tTA; TO) controls at a false discovery rate (FDR)<0.1. The minimum fold-changes corresponding to these FDRs (up- or down-regulation) were 1.16-fold (16%) for PLCβ1a/b *vs* blank virus, and 1.2-fold (20%) for Gαq *vs* wt or PKCα *vs* tet-off. In order to compare expression changes for all 781 mRNAs across all three experiments, the fold-change of each mRNA between ‘treatment’ and appropriate ‘control’ groups was computed, the mean mRNA abundance value of control hearts was adjusted to 1 (log_2_ value = 0), and the resulting variation across individual hearts from each group was displayed in a heatmap (Partek Genomics Suite v6.6, Partek, St. Louis, MO).

## Results

### Functional and transcriptional responses to PLCβ1b or PLCβ1a expression

**Fig 2:**
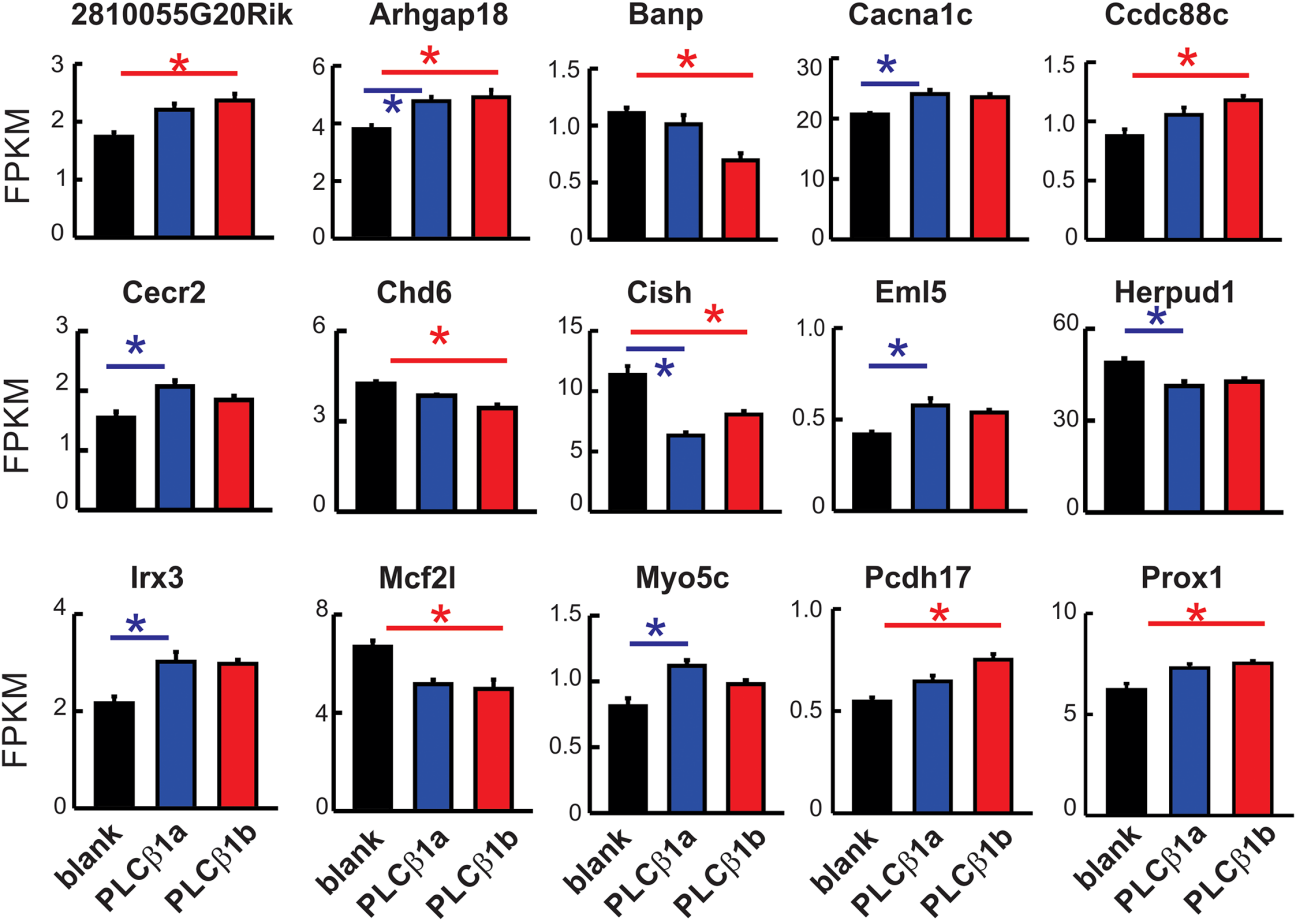
mRNAs regulated at FDR<0.1 by either or both of PLCβ1a or PLCβ1b expression. 15 mRNAs are displayed according to expression calculated from mRNA-sequencing (FPKM). *Red*, PLCβ1b; *blue*, PLCβ1a; *black*, blank control. Values are mean ± SEM, n = 6. Blue lines with asterisk indicate false discovery rate (FDR) <0.1 between PLCβ1a and blank (according to DESeq); red lines with asterisk indicate FDR <0.1 between PLCβ1b and blank. Full gene descriptions and assignment to Gene Ontology categories are given in S1 Table.

**Fig 3:**
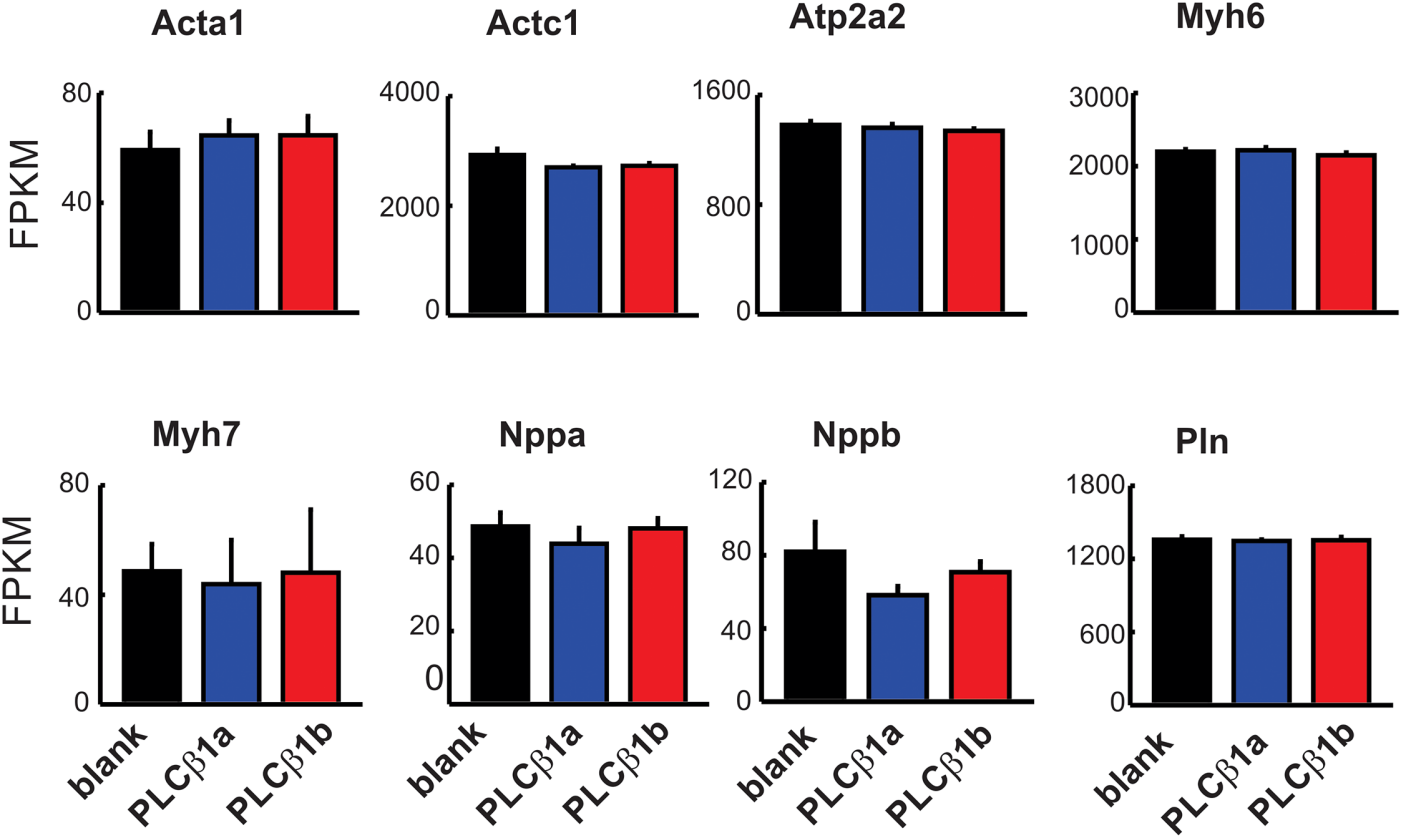
Canonical markers of cardiac hypertrophy in response to PLCβ1a or PLCβ1b expression. mRNAs are displayed according to expression calculated from mRNA-sequencing (FPKM, fragments per kilobase of exon per million mapped fragments). *Red*, PLCβ1b; *blue*, PLCβ1a; *black*, blank control. Values are mean ± SEM, n = 6. No comparisons to blank control were significant at FDR<0.1 (according to DESeq). Acta1, α-skeletal actin; Actc1, α-cardiac actin; Atp2a2, SERCA2a; Myh6, α-myosin heavy chain; Myh7, β-myosin heavy chain; Nppa, atrial natriuretic peptide; Nppb, natriuretic peptide B; Pln, phospholamban.

PLCβ1b or the splice variant that is functionally inactive in heart, PLCβ1a, were overexpressed in mouse hearts *in vivo* using rAAV6-mediated transduction (Fig 1A and 1B). Overexpression of PLCβ1b in mouse hearts for 8 weeks resulted in depressed contractile function, expressed as ejection fraction (EF%), without detectable hypertrophy. These data are similar to those reported previously and are typical of 5 different cohorts (Fig 1C, 1D and 1E; Table 1) [14]. In all cases, contractile dysfunction reached a plateau between 8–10 weeks after injection and was sustained for several months followed eventually by hypertrophy. For convenience, data from previous studies are depicted diagrammatically in Fig 1D, allowing the time frame of the current study to be seen in relation to phenotype progression. We hypothesized that low contractility and decreased cardiac output would be sufficient to trigger a compensatory transcriptional response. However, despite the low contractility in PLCβ1b-expressing mice, unbiased whole-transcriptome analysis identified only minimal differences between hearts from mice expressing PLCβ1b and hearts from mice transduced with a control vector that does not express a protein product (rAAV6-blank). Only nine (9) genes showed significant expression differences between these two groups at a false discovery rate (FDR) < 0.1 (which represents a rather lenient filter compared to typical FDRs < 0.05 or < 0.02) (Fig 2, S1 Table). Similar expression changes were observed when data from rAAV6-PLCβ1a expressing mice were compared with rAAV6-blank mice. A total of 15 genes showed a transcriptional response at FDR < 0.1 to either or both of PLCβ1b or PLCβ1a expression (Fig 2, S1 Table); these changes were all of relatively low magnitude and did not include any canonical genes known to be involved in the regulation of cardiac contractility or hypertrophy (Fig 3).

**Table 1:** Echocardiographic parameters in mice after 8 weeks of rAAV6 treatment.

	blank	PLCβ1a	PLCβ1b
LVAW_d_ (mm)	0.55 ± 0.03	0.53 ± 0.02	0.56 ± 0.03
LVAW_s_ (mm)	0.97 ± 0.03	0.98 ± 0.02	0.92 ± 0.03
LVID_d_ (mm)	3.99 ± 0.20	3.92 ± 0.13	4.01 ± 0.08
LVID_s_ (mm)	2.30 ± 0.19	2.25 ± 0.11	2.67 ± 0.07*
LVPW_d_ (mm)	0.74 ± 0.04	0.72 ± 0.02	0.72 ± 0.03
LVPW_s_ (mm)	1.35 ± 0.05	1.30 ± 0.02	1.14 ± 0.05*
HR (bpm)	548 ± 15	551 ± 5	553 ± 7
EF (%)	80.9 ± 2.1	81.0 ± 1.2	70.2 ± 1.8*
PWT (%)	85.5 ± 8.9	80.3 ± 4.5	58.8 ± 4.1*
Heart weight (g)	31.8 ± 0.6	31.1 ± 0.5	31.3 ± 0.4

Mice were treated with rAAV6-FLAG-PLCβ1b, rAAV6-FLAG-PLCβ1a or rAAV6-blank for 8 weeks and contractile function was determined by echocardiography. LVAW, left ventricular anterior wall thickness; LVID, left ventricular interior dimension; LVPW, left ventricular posterior wall thickness; HR, heart rate; EF, ejection fraction; PWT, posterior wall thickening index. Subscript s and d represent left ventricular measurements at systole ‘s’ or diastole ‘d’. Values shown are mean ± SEM, n = 6. Similar results were observed in 4 additional cohorts.

*p<0.05 relative to either blank or PLCβ1a control.

Statistical significance between groups determined using one-way ANOVA (Tukey’s post-hoc test). Although similar data have been previously reported as part of longer time course studies (14) data from the current cohort of mice are presented here for convenience in understanding the phenotype of these mice 8 weeks after rAAV6 treatment.

Since mRNA-sequencing studies utilizing genetically- and/or surgically-manipulated mouse hearts with similar numbers of biological replicates have typically observed hundreds of altered mRNAs [24–26, 28, 29], we evaluated the variance to read-count relationship for the mRNAs subjected to statistical comparison and found that variance declined for mRNAs with higher read counts in the expected manner (S1 Fig). This suggests that the extremely modest transcriptional response observed in rAAV6-PLCβ1a/b mouse hearts is not due to technical artefacts in the mRNA-sequencing procedure.

### Comparison of PLCβ1b transcriptional responses to those resulting from neonatal induction of upstream and downstream signal transducers

**Fig 4:**
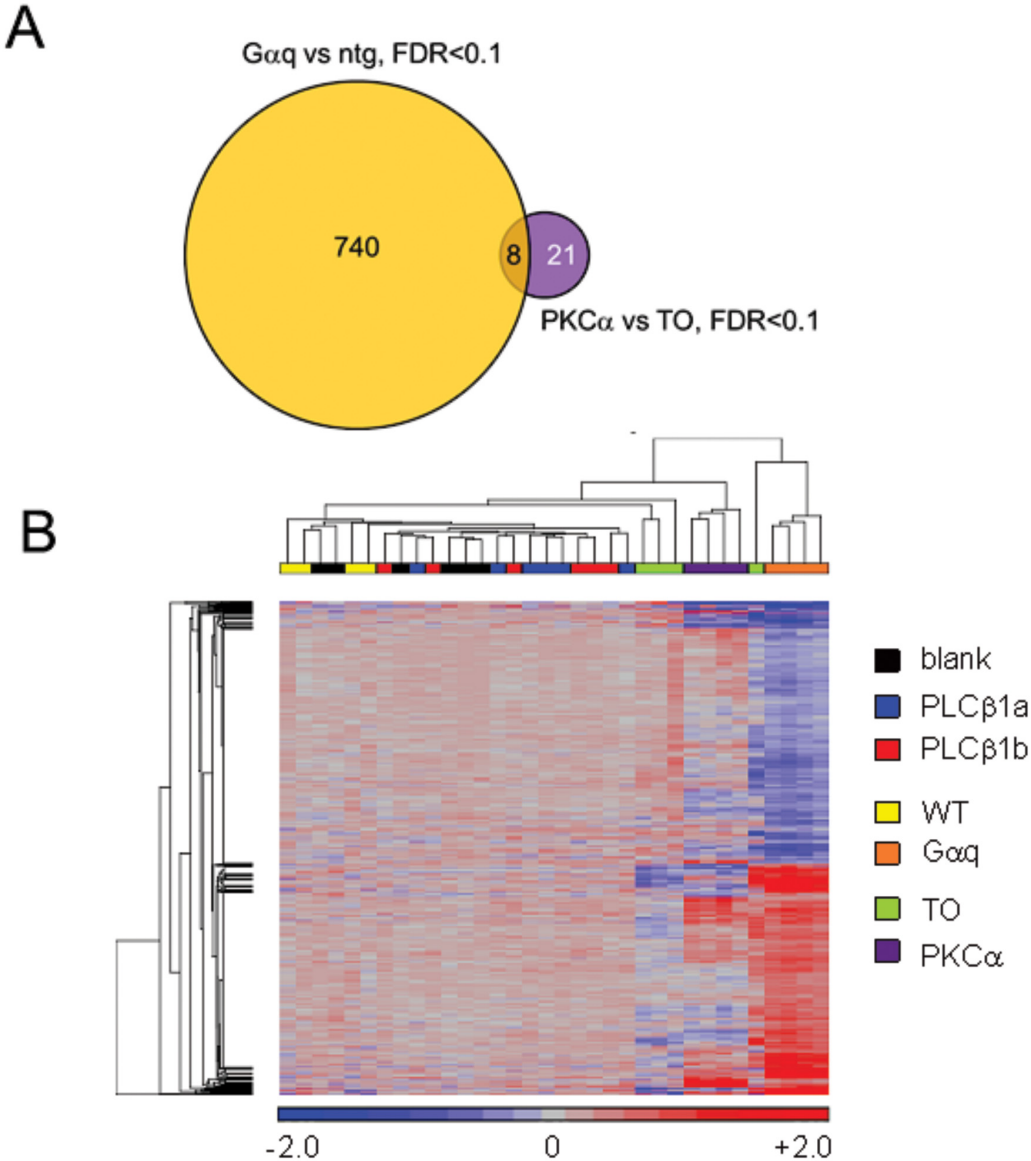
Comparison of rAAV6-PLCβ1-mediated gene expression changes to those caused by neonatal overexpression of Gαq or PKCα. **A**. Venn diagram demonstrating limited overlap of 748 mRNAs regulated at FDR<0.1 in a comparison of Gαq transgenic mice vs WT [24] and 29 mRNAs regulated at FDR<0.1 in a comparison of PKCα transgenic mice vs tet-off (TO) controls [27]. **B**. 761 unique mRNAs are displayed, from a composite of 15 mRNAs regulated at FDR<0.1 in a comparison of PLCβ1a or PLCβ1b vs blank, 748 mRNAs regulated at FDR<0.1 in a comparison of Gqα transgenic mice vs WT [24] and 29 mRNAs regulated at FDR<0.1 in a comparison of PKCα transgenic mice vs tet-off (TO) controls [27]. Unsupervised hierarchical clustering of relative mRNA abundances (rows) and of individual hearts (columns) was performed using Euclidean distance with average linkage. Colors represent log_2_ ratios for each individual heart vs the mean of the appropriate controls (red is upregulated, blue is downregulated); the color range spans -2.0 to +2.0 in log_2_ scale (from 4-fold downregulation to 4-fold upregulation in linear scale).

Cardiac overexpression of either Gαq (upstream regulator of PLCβ1b [16]) or of PKCα (activated downstream of PLCβ1b [14]) beginning in the immediate post-natal period are known to induce widespread transcriptional responses, and in the case of Gαq a well-described phenotype of hypertrophy and dilation with severely blunted contractility occurs as a result [30–32]. As might be expected from the phenotypic outcomes, many more mRNAs are dysregulated in adult hearts overexpressing Gαq than PKCα, in which transgene expression (under the control of the αMHC / *Myh6* promoter) began immediately after birth (see Methods) (Fig 4A) [24, 27]. We compared the transcriptomes of rAAV6-PLCβ1a and -PLCβ1b hearts (without filtering for statistical significance of differentially-expressed mRNAs) to published mRNA-seq profiles of adult hearts overexpressing Gαq or PKCα from the post-natal period [24, 27]. However, neither PLCβ1a nor PLCβ1b hearts showed any evidence of Gαq- or PKCα-mediated alterations in transcriptional profile (Fig 4B).

## Discussion

Our previous studies showed that heightened expression of PLCβ1b is sufficient to cause chronic contractile dysfunction, whereas expressing the closely related splice variant PLCβ1a had no discernible effect [14]. Furthermore, selectively inhibiting PLCβ1b activation prevented/reversed heart failure following pressure overload [15]. Hearts expressing PLCβ1b showed depressed contractile function without hypertrophy or fibrosis, at least until 36 weeks after virus delivery. This provided an opportunity to examine whether transcriptional responses occur in response to loss of contractility in isolation, without the difficulty of disentangling these from responses associated with hypertrophied or failed myocardium. PLCβ1b presented an especially attractive opportunity because PLCβ1a has no effect on contractility and could thus serve as a stringent control.

Despite chronically lowered contractility, we detected only minor changes in gene transcription associated with PLCβ1b expression and furthermore, similar changes followed expression of PLCβ1a, even though PLCβ1a did not alter contractile function. Thus, depressed contractility *per se* does not lead to widespread compensatory transcriptional changes. Our previous report noted changes in canonical transcriptional markers of cardiac hypertrophy, once hypertrophy finally ensued in PLCβ1b hearts, presumably as a result to prolonged contractile depression. After only 8 weeks of chronically lowered contractility, we found no changes in the expressions of ANP and α-skeletal actin, collagen-1A1, -3A1, α-or β-myosin heavy chain or phospholamban as measured by RT-qPCR [14], nor did we in the current RNA-sequencing study. After 32 weeks of depressed contractility, PLCβ1b-expressing hearts showed a modest degree of hypertrophy. At this time point, increased expression of ANP and α-skeletal actin was observed in PLCβ1b-expressing hearts compared with either PLCβ1a-expressing hearts or control hearts [14].

The finding that PLCβ1b-induced contractile dysfunction does not involve substantial transcriptional changes is compatible with our previous data, which showed that the contractile deficit could be fully reversed by treatment with a PKCα inhibitor for only 5 days [14, 33]. This rapid reversal argues against an involvement of major transcriptional changes in the contractile dysfunction. In addition, our studies in adult mouse ventricular myocytes and neonatal rat ventricular myocytes showed that PLCβ1b overexpression results in dephosphorylation of phospholamban and depletion of Ca^2+^ from the sarcoplasmic reticulum [14]. Neither of these responses would be expected to require altered gene transcription, as they are likely dependent on well-established phosphorylation cascades [34]. However, it is perhaps surprising that chronically low contractility and reduced cardiac output does not of itself induce any substantial transcriptional response to increase inotropy or lusitropy. Presumably the hypertrophy that eventually ensues is such a response.

Whilst we conclude that chronically depressed contractility does not meaningfully alter transcription, it must be considered that the PLCβ1b-expressing mice were unchallenged, caged and fed and so had limited requirement for flight or fight responses. It remains possible that the combination of chronically low contractility induced by PLCβ1b and a stressor requiring increased cardiac performance might result in an altered transcription response compared with stressed but otherwise untreated mice. As a result, stressing PLCβ1b-overexpressing mice might result in a more rapid development of compensatory hypertrophy, together with its associated transcriptional changes.

In cardiac signal transduction, PLCβ1b is immediately downstream of Gαq [16] and upstream of PKCα [14]. Our previous data have provided evidence that PLCβ1b is specifically activated downstream of Gαq in heart and thus similar transcriptional responses might be expected. In neonatal rat ventricular myocytes, the phenotype caused by heightened expression of Gαq is similar to that caused by PLCβ1b [16], with either of these causing primarily a hypertrophic response. However, both the phenotype and the transcriptional response to Gαq in mouse hearts are dependent on the timing of Gαq overexpression. Use of the conventional αMHC promoter system for Gαq transgene expression beginning immediately after birth promotes hypertrophy, dilatation and failure [30–32] and this is associated with substantial changes in the transcriptome [26]. In marked contrast, conditional overexpression of Gαq initiated in adult mouse hearts (a condition more similar to the rAAV6-mediated delivery used in our studies) did not result in hypertrophy, dilatation or heart failure and the limited mRNA expression data provided showed no changes in canonical markers of cardiac hypertrophy [35]. Whilst comprehensive transcriptome data are unfortunately not available for this latter condition, it seems unlikely that widespread transcriptional changes would be present in hearts exhibiting normal function. As noted previously [14], functional responses of the mouse heart to heightened PLCβ1b are similar to those reported previously in PKCα-overexpressing mice [36] and our data are consistent with a signaling role for PLCβ1b upstream of PKCα in mouse heart [14]. A relatively small set of mRNA expression changes was observed in PKCα-expressing hearts even though PKCα transgene expression commenced at birth [5, 27, 36]. Thus, findings from transgenes expressed from the immediate post-natal period are not directly comparable with the current data where heightened expression was initiated in adult animals. This also speaks to the importance of evaluating phenotypes in which signaling has been altered concomitant with the typical post-natal enlargement of the heart and acquisition of adult metabolic and sarcomeric properties, in contrast to phenotypes in which signaling is only altered once the heart has fully matured.

In addition to showing that depressed contractility *per se* does not initiate major transcriptional responses, our study also stresses the importance of a stringent control group in studies of this type. Although transcriptional changes were detected in response to PLCβ1b expression, these do not appear intuitively connected to pathways known to cause, or to be affected as a consequence, of blunted contractility. Because these alterations were similar to those in PLCβ1a-expressing hearts, we were able to define these as events not specific to PLCβ1b overexpression, but caused by the overexpression of the common molecular domains of PLCβ1a and PLCβ1b, which are 97% homologous (Fig 1A) and differ only in membrane-targeting capacity [37].

We have developed a mini-gene inhibitor that specifically targets PLCβ1b activation by preventing its targeting to the sarcolemma and have shown that expressing this inhibitor in hearts protects from heart failure following pressure overload. Additionally, we have identified the binding interface responsible for sarcolemmal targeting, by showing that proline-rich sequences in the extreme C-terminal sequence of PLCβ1b bind to the SH3 domain of the scaffolding protein Shank3 [37]. This binding interface provides a potential therapeutic target for the development of a new class of inotropic agent. The finding that PLCβ1b does not induce transcriptional changes makes targeting its activation a more attractive proposition for drug development.

## Supporting Information

S1 Fig Variance to read-count relationship for the mRNAs subjected to statistical comparison. Dispersion (variance) declines in accordance with increasing read depth-normalized number of counts per mRNA.
(TIF)

S1 Table Gene descriptions and assignment to Gene Ontology categories for the 15 mRNAs regulated at FDR<0.1 by rAAV6-PLCB1a or rAAV6-PLCB1b expression. Assignment to Gene Ontology ‘biological process’ categories was performed using the Cytoscape plugin BiNGO [1]. Chd6 (chromodomain helicase DNA binding protein 6) had no Gene Ontology entry and is therefore not listed.
(XLS)
