# Supplementary figures and images for: Chronic Contractile Dysfunction without Hypertrophy Does Not Provoke a Compensatory Transcriptional Response in Mouse Hearts
Source: PLoS One. 2016 Jun 30;11(6):e0158317. doi: 10.1371/journal.pone.0158317 (PMC4928941; doi:10.1371/journal.pone.0158317)

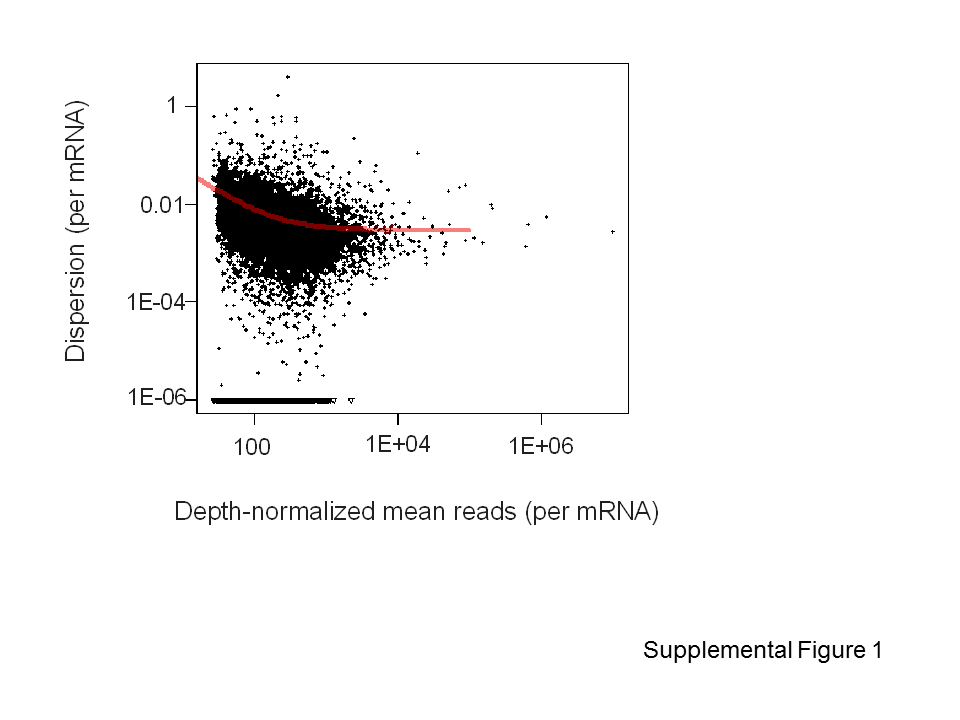

Supplement: S1 Fig — Dispersion (variance) declines in accordance with increasing read depth-normalized number of counts per mRNA. (TIF) [file pone.0158317.s001.tif]
